# Depression and sexual risk behavior among long-distance truck drivers at roadside wellness clinics in Kenya

**DOI:** 10.7717/peerj.7253

**Published:** 2019-07-18

**Authors:** Matthew L. Romo, Gavin George, Joanne E. Mantell, Eva Mwai, Eston Nyaga, Michael Strauss, Jacob O. Odhiambo, Kaymarlin Govender, Elizabeth A. Kelvin

**Affiliations:** 1 Department of Epidemiology and Biostatistics & Institute for Implementation Science in Population Health, Graduate School of Public Health and Health Policy, City University of New York, New York, NY, USA; 2 School of Public Health, Li Ka Shing Faculty of Medicine, The University of Hong Kong, Hong Kong, Hong Kong SAR; 3 Health Economics and HIV and AIDS Research Division, University of KwaZulu-Natal, Durban, South Africa; 4 HIV Center for Clinical and Behavioral Studies, Department of Psychiatry, Division of Gender, Sexuality, and Health, New York State Psychiatric Institute & Columbia University Medical Center, New York, NY, USA; 5 North Star Alliance, Nairobi, Kenya

**Keywords:** Kenya, Mental health, Depressive disorder, Sexual behavior, Self efficacy

## Abstract

**Background:**

Truck drivers in sub-Saharan Africa are at high risk for both mental health disorders and sexually transmitted infections. We sought to determine the prevalence of depression among a sample of long-distance truck drivers seeking services at roadside wellness clinics in Kenya and explore the relationship between depression and sexual risk behavior.

**Methods:**

We used data from an interviewer-administered questionnaire from 284 truck drivers in Kenya who participated in a randomized controlled trial evaluating whether offering oral HIV self-testing could increase HIV test uptake. Depression was categorized based on the Patient Health Questionnaire-9 score, with a score ≥10 indicative of probable major depressive disorder (MDD). Sexual risk behavior was operationalized as the number of condomless sex partners in the past 6 months.

**Results:**

The mean participant age was 36.9 years, 83.0% were married, and 37.0% had a secondary school education or higher. Overall, 24% of participants had probable MDD, and 58.2% reported having one condomless sex partner in the past 6 months, whereas 27.3% reported having had two or more. In a multivariable Poisson regression model adjusted for demographic and other relevant variables, including number of sex partners, MDD was significantly associated with a greater number of condomless sex partners (adjusted prevalence ratio 1.63, 95% confidence interval [1.25–2.12], *p* < 0.001). General self-efficacy significantly mediated the association between MDD and number of condomless sex partners.

**Conclusions:**

The high prevalence of depression highlights the need to test the feasibility and acceptability of mental healthcare interventions for this population, possibly integrated with HIV prevention services. Future research is needed to better understand the association between depression and sexual risk behavior, as well as the role of self-efficacy.

## Introduction

Few published studies have comprehensively explored the epidemiology of depression among truck drivers and none, to our knowledge, have done so in sub-Saharan Africa. Long-distance truck drivers may have a high prevalence of depression (and possibly other mental health disorders) due to occupational related stressors (Melchior et al., 2007; Munce et al., 2006; Wang, 2005; Wulsin et al., 2014), such as road hazards, fixed delivery deadlines, and irregular sleeping schedules (Shattell et al., 2010). Globally, several studies have estimated the prevalence of depression among truck drivers, but these estimates have varied by country, ranging from 14% to 70% (Da Silva-Júnior et al., 2009; Fan et al., 2012; Shattell et al., 2012; Rice et al., 2016; Shen et al., 2013; Vakili et al., 2010).

Truck drivers are also at high-risk of HIV infection because of the high rates of sexual risk behavior, including engaging in condomless sex and having multiple partners (Costenbader et al., 2015; Morris & Ferguson, 2007; Yaya et al., 2016; Botao et al., 2012). A small number of studies in sub-Saharan Africa have explored the relationship between depression and sexual risk, including in a general population at risk for HIV infection in South Africa (Smit et al., 2006; Nduna et al., 2010) and among social networks of young men in Tanzania (Hill et al., 2016). Although this relationship is complex, one possible explanation may be that maladaptive thought patterns arising from depression, such as those related to impulsivity and hopelessness (Wang et al., 2015), might lead to the adoption of risky behavior and increase the propensity for self-harm. In other words, depression might lead to increased sexual risk behavior. General self-efficacy, which can be defined as “people’s belief in their ability to influence events that affect their lives” (Bandura, 2010), might mediate this association, as suggested by previous studies in sub-Saharan Africa (Wagner et al., 2011; Alvy et al., 2011; Tucker et al., 2013).

In this study, we assessed the prevalence of depression and explored its association with sexual risk behavior in a sample of long-distance truck drivers seeking services at two roadside wellness clinics in Kenya. Additionally, we explored whether general self-efficacy mediated the association between depression and sexual risk behavior.

## Materials and Methods

### Study procedures

We used cross-sectional data collected as part of a randomized controlled trial evaluating whether offering HIV testing options (i.e., provider-administered blood-based (finger prick) rapid HIV test or a supervised oral self-administered rapid HIV test (OraQuick In-Home HIV Test) in the clinic or a self-test kit for home use) compared with the standard of care (i.e., only offering the provider-administered blood test) would increase HIV testing rates among truck drivers in Kenya. The methods and results of this trial are described in detail elsewhere (Kelvin et al., 2018).

Participants were recruited from two North Star Alliance roadside wellness clinics located along the northern transport corridor in Nakuru County, Kenya, one of the highest HIV prevalence areas in the country (National AIDS Control Council, 2014; Kenya Ministry of Health, 2014). The North Star Alliance provides healthcare services, including sexually transmitted infection and HIV testing and treatment (North Star Alliance, 2015), to key populations in transport corridor communities, including truck drivers and sex workers. At the time of this study, the North Star Alliance had 36 clinics in Africa, eight of which were in Kenya. Together, the two clinics included in this study served about 400 clients weekly, 30% of whom were long-distance truck drivers. Any male truck driver who visited either of the two clinics from October to December 2015 for services other than HIV treatment, was informed about the research study and, if interested, was referred to a fieldworker for information and eligibility screening. Eligibility criteria were: (1) at least 18 years old, (2) male (based on observation), (3) employed as a long-distance truck driver (anyone who works to make deliveries locally would be classified as a community resident and not eligible for our study), (4) primary residence in Kenya, (5) able to speak English or Kiswahili, (6) self-reported HIV-negative or unknown HIV status, (7) able to sign the consent form, and (8) willing to receive payment for participation via MPesa, a cell phone-based money transfer system widely used in Kenya. Overall, 319 men were referred for eligibility screening, of whom 305 were enrolled in the study. Informed consent was obtained from all participants.

Fieldworkers administered a baseline interview to truck drivers who met the eligibility criteria and consented to participate in the study. The baseline interview included questions about demographic characteristics and risk behavior, and several psychometric scales, including general self-efficacy. Following the baseline interview, participants were offered HIV testing with options dependent on randomization arm (i.e., choice of an alternative HIV testing modality vs. standard provider-administered blood test). After undergoing or refusing HIV testing, appointments were made for a follow-up interview at 6 months. At the 6-month interview, which was completed over the phone in most cases, research staff administered the Patient Health Questionnaire (PHQ)-9 (Kroenke, Spitzer & Williams, 2001) and asked about HIV testing in the past 6 months. The questionnaires were developed in English and Kiswahili, and the Kiswahili translation was translated and back-translated to English to ensure accuracy. Interviews were conducted in Kiswahili, English, or both, depending on the participant’s preference. The study procedures were approved by the City University of New York Institutional Review Board (#2015-0645), the Kenya Medical Research Institute Ethics Committee (#541), and the University of KwaZulu-Natal Biomedical Research Ethics Committee (BFC025/15), and were in accordance with the 1964 Helsinki declaration and its later amendments.

### Measures

#### Sexual risk behavior (main outcome of interest)

Number of condomless sex partners was used as a measure of sexual risk behavior and was determined by asking participants how many female and male condomless sex partners they had in the past 6 months. Participants who did not have sex in the past 6 months (*n* = 6) were categorized as having zero condomless sex partners. Only one reported ever having anal sex with another man. We retained this variable as a numeric, but also categorized responses (none, one, two or more) in bivariate analyses.

#### Depression (main exposure of interest)

Depression was assessed with the PHQ-9, a widely used and validated nine-item questionnaire asking about each Diagnostic and Statistical Manual of Mental Disorders-IV depressive symptom within the last 2 weeks (Kroenke, Spitzer & Williams, 2001). Each item is scored based on symptom frequency, from 0 (“not at all”), 1 (“several days”), 2 (“more than half the days”), or 3 (“nearly every day”), with scores can ranging from 0 to 27. The PHQ-9 has been formally validated among general adult populations using an acceptable gold standard (i.e., psychiatric clinician diagnosis using Schedules for Clinical Assessment in Neuropsychiatry or Mini International Neuropsychiatric Interview) in East African primary healthcare settings (Gelaye et al., 2013; Hanlon et al., 2015). We constructed a summary score by summing responses to the nine questions allowing up to one item to be left unanswered; only two participants had any missing responses, and for these two participants, only one item was missing, so no one was excluded. The standardized Cronbach’s alpha for the PHQ-9 in our study sample was 0.88, indicating good to excellent internal consistency. Scores were categorized as no/minimal depression (score 0–4), mild depression (score 5–9), and moderate-severe depression (score ≥10). A score of ≥10 has 88% sensitivity and specificity for major depressive disorder (MDD) (Kroenke, Spitzer & Williams, 2001).

#### Demographic characteristics, self-efficacy, and other risk behavior

In the baseline interview, participants were asked their age, religion, educational attainment, and marital status. Age was based on reported age at their last birthday (in years). Religion was categorized as Christian (i.e., Protestant, Catholic) or non-Christian (i.e., Muslim, Hindu, traditional African, and no religion). Educational attainment was categorized as having completed at least secondary school vs. less than a secondary school diploma (i.e., no education, some primary school, completed primary school only, or some secondary school). Marital status was categorized as currently married (either legal or common-law) vs. unmarried (including divorced/separated, widowed, or single).

Average monthly income from truck driving was determined based on responses to the question “about how much money do you earn in an average month driving a truck?” and those who were unable or unwilling to specify their income were then asked “could you tell me if your income is less than 8,000 Kenyan shillings (Ks), 8,000–16,000, 16,001–24,000, 24,001–50,000, or >50,001 Ks?” At the time of the study, 100 Ks was worth approximately 1.00 US dollar (USD). Income was dichotomized at about the first quartile into mid-high income (≥240 USD) vs. low income (<240 USD). Length of time worked as a truck driver was determined by asking participants how many months and years they had worked as a truck driver. Driving accompanied or not was determined by asking participants if they usually drive alone or if someone else rides with them (e.g., assistant, main partner, non-main partner, family member, etc.). Days away from home in the past month was determined by asking participants how many nights they were away from home traveling for work in the past month.

General self-efficacy was measured using a 10-item scale (Schwarzer & Jerusalem 1995), previously validated in a general non-white South African population (Keyes et al., 2008), which presents statements related to belief in one’s confidence to cope with a broad range of stressful or challenging demands. Response options were on a four-point Likert scale from “not at all true” to “exactly true,” with a possible total score range of 10–40. We constructed a summary score by summing responses to the 10 questions allowing for one of the items to be left unanswered; only three participants had any missing responses and they were missing only one item. Cronbach’s alpha for this scale in our sample was 0.89, indicating good to excellent internal consistency.

Number of sex partners was determined by asking how many people they had sexual intercourse with in the past 6 months, asking separately for female (vaginal/anal intercourse) and male (anal intercourse) partners. Alcohol use was determined by asking how often the participant had drinks containing alcohol in the past year. Similarly, illicit drug use was determined by asking how often the participant used drugs in the past year, with marijuana and cocaine listed as examples. Response options for both questions were “at least once a day,” “a few times a week but not every day,” “a few times a month but not every week,” “a few times a year but not every month,” and “never.” We recoded these variables into two separate indicators for having consumed any alcohol or drugs in the past year (both versus none) for bivariate analyses. However, as there were very few reports of drug use and all participants who reported drug use also reported alcohol use, only the alcohol use variable was included in the regression models.

### Statistical analysis

We first described the sample overall and by depression category. We then used bivariate statistical tests (Pearson’s Chi-square, Fisher’s exact, and Kruskal–Wallis) to identify significant differences in the distribution of variables by depression category. We then ran Poisson regression models with number of condomless sex partners as the dependent variable. We ran crude models, then a multivariable model that included all variables explored in bivariate analyses (with the addition of clinic of enrollment). Next, we ran a multivariable model with imputed data to address potential bias due to missing data. The multivariable model had about 25% missing data, so we ran 25 imputations to achieve reliable *p*-values and standard errors (Bodner, 2008).

We used fully conditional specification for the imputation method (logistic for classification variables and predictive mean matching for numeric variables) (Berglund & Heeringa, 2014) with all variables in the model used to impute missing values. We then re-ran our multivariable model with the imputed data.

Finally, we assessed whether general self-efficacy mediated the association between MDD (PHQ-9 score ≥10) and number of condomless sex partners. This analysis was conducted using a SAS Macro developed by Valeri & VanderWeele (2013), with mediation methodology from Pearl (2014). The Macro used a bootstrapping approach with 100 samples to generate crude prevalence ratios (cPRs) and 95% confidence intervals (CIs) for the natural direct effect and natural indirect effect of the association between MDD and number of condomless sex partners (Valeri & VanderWeele, 2013). Presence of a statistically significant natural indirect effect was evidence of mediation.

All analyses were conducted in SAS 9.4 (SAS Institute Inc., Cary, NC, USA) and the significance level was set at a two-sided α of 0.05.

## Results

### Description of the sample

The mean age of the overall sample was 36.9 years (standard deviation (SD) = 7.9). Most participants were married (83.0%), about one-third (37.0%) had a secondary school education or greater, about three-quarters (77.8%) were Christian, and nearly three-quarters (73.5%) earned ≥240 USD monthly. Participants had worked as a truck driver on average for 8.8 years (SD = 7.0), more than half (58.1%) usually drove alone, and in the previous 30 days, participants spent a mean of 21.6 days (SD = 23.0) on the road. Mean general self-efficacy score was 36.5 (SD = 4.6). In terms of risk behaviors, 53.5% had consumed alcohol in the past year and 3.2% reported drug use in the past year. The mean number of sex partners in the past 6 months was 2.8 (SD = 4.1); 58.2% reported having at least one condomless sex partner in the past 6 months and 27.3% reported having two or more (Table 1).

**Table 1:** Demographic variables, risk behaviors, and other characteristics overall and by depression category.

	All participants (with depression data)	None/minimal depression	Mild depression	Moderate-severe depression	*p*-Value
*n* (%)	284 (100%)	157 (55.3%)	59 (20.8%)	68 (23.9%)	NA
Age (years)	*N* = 284				
Mean (SD)/Median (IQR)	36.9 (7.9)/36.0 (10.0)	36.9 (8.0)/37.0 (10.0)	37.0 (7.0)/37.0 (9.0)	36.8 (8.5)/35.0 (11.5)	0.859^a^
Married	*N* = 282				
Yes, *n* (%)	234 (83.0%)	130 (55.6%)	50 (21.4%)	54 (23.1%)	0.660^b^
No, *n* (%)	48 (17.0%)	25 (52.1%)	9 (18.8%)	14 (29.2%)	
Secondary school education or higher	*N* = 284				
Yes, *n* (%)	105 (37.0%)	57 (54.3%)	25 (23.8%)	23 (21.9%)	0.589^b^
No, *n* (%)	179 (63.0%)	100 (55.9%)	34 (19.0%)	45 (25.1%)	
Religion	*N* = 279				
Christian, *n* (%)	217 (77.8%)	117 (53.9%)	47 (21.7%)	53 (24.4%)	0.436^b^
Non-Christian, *n* (%)	62 (22.2%)	39 (62.9%)	10 (16.1%)	13 (21.0%)	
Monthly income	*N* = 268				
<240 USD, *n* (%)	71 (26.5%)	34 (47.9%)	10 (14.1%)	27 (38.0%)	0.010^b^
≥240 USD, *n* (%)	197 (73.5%)	112 (56.9%)	45 (22.8%)	40 (20.3%)	
Years worked as a truck driver	*N* = 284				
Mean (SD)/Median (IQR)	8.8 (7.1)/7.0 (7.0)	9.2 (6.5)/8.0 (8.0)	9.3 (8.0)/6.7 (6.8)	7.6 (7.4)/4.2 (6.3)	0.016^a^
Usually drives alone	*N* = 284				
Yes, *n* (%)	165 (58.1%)	97 (58.8%)	32 (19.4%)	36 (21.8%)	0.372^b^
No, *n* (%)	119 (41.9%)	60 (50.4%)	27 (22.7%)	32 (26.9%)	
Days away on road in past 30 days	*N* = 277				
Mean (SD)/Median (IQR)	21.6 (5.5)/23.0 (5.0)	21.5 (5.6)/22.0 (5.0)	21.7 (5.4)/22.0 (5.0)	21.9 (5.5)/24.0 (5.0)	0.766^a^
General self-efficacy (score)	*N* = 284				
Mean (SD)/Median (IQR)	36.5 (4.6)/39.0 (5.0)	38.2 (2.9)/40.0 (3.0)	35.3 (5.3)/37.0 (8.0)	33.7 (5.6)/36.0 (10.5)	<0.001^a^
Alcohol use in past year^d^	*N* = 284				
Yes, *n* (%)	152 (53.5%)	79 (52.0%)	38 (25.0%)	35 (23.0%)	0.168^b^
No, *n* (%)	132 (46.5%)	78 (59.1%)	21 (15.9%)	33 (25.0%)	
Drug use in past year^d^	*N* = 283				
Yes, *n* (%)	9 (3.2%)	0 (0.0%)	1 (11.1%)	8 (88.9%)	<0.001^c^
No, *n* (%)	274 (96.8%)	129 (47.1%)	86 (31.4%)	59 (21.5%)	
Number of sex partners in past 6 months	*N* = 268				
Mean (SD)/Median (IQR)	2.8 (4.1)/2.0 (3.0)	2.6 (5.4)/2.0 (2.0)	2.4 (1.7)/2.0 (2.0)	3.5 (3.5)/2.0 (4.0)	0.085^a^
Number of condomless sex partners in past 6 months	*N* = 275				
Mean (SD)/Median (IQR)	1.5 (1.6)/1.0 (1.0)	1.2 (1.1)/1.0 (0.0)	1.3 (1.1)/1.0 (0.0)	2.2 (2.6)/1.0 (2.0)	0.003^a^
None, *n* (%)	40 (14.6%)	23 (57.5%)	11 (27.5%)	6 (15.0%)	0.038^b^
One, *n* (%)	160 (58.2%)	73 (45.6%)	54 (33.8%)	33 (20.6%)	
Two or more, *n* (%)	75 (27.3%)	27 (36.0%)	21 (28.0%)	27 (36.0%)	

**Notes:**

Some percentages may not add up to 100% due to rounding.

IQR, interquartile range; SD, standard deviation; USD, US dollars.

a Kruskall–Wallis test.

b Pearson’s Chi-square test.

c Fisher’s exact test.

d All participants who reported drug use also reported alcohol use. Drug use was not included in the regression models because of small numbers of participants in categories.

The mean and median scores on the PHQ-9 were 5.2 (SD = 5.0) and 4.0 (interquartile range = 9.0), respectively. Upon categorizing participants, 157 (55.3%) had none/minimal depression, 59 (22.8%) had mild depression, and 68 (24.0%) had moderate-severe depression (probable MDD) (Table 1). The prevalence of any symptom frequency in the past 2 weeks based on the PHQ-9 was: low energy (50.7%), trouble concentrating (50.4%), anhedonia (little interest or pleasure in doing things) (48.8%), appetite problems (44.2%), feeling depressed (40.8%), sleep problems (41.2%), low self-esteem (38.3%), psychomotor problems (30.6%), and suicide/self-harm ideation (19.4%).

### Bivariate analysis results

In bivariate analyses, there were significant differences in the distribution of monthly income, years worked as a truck driver, general self-efficacy, drug use, and number of condomless sex partners by depression category. Participants with lower monthly trucking income (<240 USD) vs. higher monthly trucking income (≥240 USD) more often had moderate-severe depression (38.0% vs. 20.3%; *p* = 0.010). Truck drivers with moderate-severe depression vs. no/minimal depression tended to have been in the occupation for a shorter period of time (mean years: 7.6 vs. 9.2; *p* = 0.016). General self-efficacy scores were lower for participants with depression (mean score 38.2 (none/minimal depression), 35.3 (mild depression), 33.7 (moderate-severe depression); *p* < 0.001). Participants who reported drug use were more likely to have depression (0.0% (no/minimal depression), 11.1% (mild depression), and 88.9% (moderate-severe depression)). Participants with moderate-severe depression were also more likely to have more condomless sex partners vs. participants with no/minimal depression (mean number of partners: 2.2 vs. 1.2; *p* = 0.003) (Table 1).

### Poisson regression results

In the crude Poisson regression model, moderate-severe depression (vs. none/minimal depression) was significantly associated with the number of condomless sex partners (cPR 1.80, *p* < 0.001). This remained significant in the multivariable Poisson regression model (adjusted prevalence ratio (aPR) 1.63, *p* < 0.001). For this model, the Pearson Chi-square value divided by the degrees of freedom was 0.92, indicating that under- and over-dispersion were not present. A goodness-of-fit Chi-squared test was not significant (*p* = 0.814), indicating reasonable model fit. In the multivariable model with imputed data, the associations between depression and condomless sex partners remained similar (mild depression: aPR 1.22, *p* = 0.124; moderate-severe depression: aPR 1.62, *p* < 0.001) (Table 2).

**Table 2:** Poisson regression models examining the association of depression and other variables with number of condomless sex partners.

	Crude models^a^	Multivariable model^b^	Multivariable model with imputed data^b^
	cPR (95% CI); *p*-value	aPR (95% CI); *p*-value	aPR (95% CI); *p*-value
		*N* = 236	*N* = 305
Depression	*N* = 275		
None/minimal	Reference	Reference	Reference
Mild	1.08 (0.84–1.37); *p* = 0.563	1.16 (0.88–1.52); *p* = 0.290	1.22 (0.95–1.56); *p* = 0.124
Moderate-severe	1.80 (1.43–2.26); *p* < 0.001	1.63 (1.25–2.12); *p* < 0.001	1.62 (1.26–2.08); *p* < 0.001
Age (years)	*N* = 296		
	1.00 (0.99–1.01); *p* = 0.817	1.01 (0.99–1.03); *p* = 0.720	1.01 (0.99–1.02); *p* = 0.360
Married	*N* = 293		
Yes	1.58 (1.17–2.12); *p* = 0.003	2.12 (1.41–3.19); *p* < 0.001	1.67 (1.20–2.32); *p* = 0.003
No	Reference	Reference	Reference
Secondary school education or higher	*N* = 296		
Yes	0.69 (0.57–0.85); *p* < 0.001	0.79 (0.61–1.02); *p* = 0.065	0.73 (0.58–0.92); *p* = 0.007
No	Reference	Reference	Reference
Religion	*N* = 290		
Christian	Reference	Reference	Reference
Non-Christian	1.26 (1.02–1.56); *p* = 0.032	1.37 (1.02–1.82); *p* = 0.034	1.34 (1.03–1.73); *p* = 0.029
Monthly trucking income	*N* = 279		
<240 USD	Reference	Reference	Reference
≥240 USD	0.64 (0.52–0.77); *p* < 0.001	0.76 (0.59–0.97); *p* = 0.029	0.72 (0.58–0.90); *p* = 0.003
Years worked as a truck driver	*N* = 294		
	0.98 (0.97–1.00); *p* = 0.010	0.98 (0.96–1.00); *p* = 0.055	0.98 (0.96–1.00); *p* = 0.017
Usually drives alone	*N* = 296		
Yes	1.43 (1.19–1.72); *p* < 0.001	1.16 (0.92–1.47); *p* = 0.220	1.10 (0.89–1.34); *p* = 0.383
No	Reference	Reference	Reference
Nights away on road in past 30 days	*N* = 288		
	0.99 (0.97–1.01); *p* = 0.183	0.98 (0.96–1.00); *p* = 0.106	0.99 (0.98–1.01); *p* = 0.475
Alcohol use in past year	*N* = 296		
Yes	1.14 (0.95–1.38); *p* = 0.158	1.11 (0.87–1.42); *p* = 0.400	1.06 (0.86–1.31); *p* = 0.593
No	Reference	Reference	Reference
Number of sex partners in the past 6 months	*N* = 288		
	1.04 (1.03–1.05); *p* < 0.001	1.04 (1.03–1.05); *p* < 0.001	1.04 (1.03–1.06); *p* < 0.001

**Notes:**

aPR, adjusted prevalence ratio; cPR, crude prevalence ratio; USD, US dollars.

a Not adjusted for any other variables.

b Adjusted for clinic location in addition to the other variables listed.

### Mediation analysis results

In the mediation analysis for general self-efficacy, the natural direct effect was not statistically significant (cPR 1.40; 95% CI [0.94–1.90]) and the natural indirect effect was significant (cPR 1.24; 95% CI [1.14–1.44]) for the association between moderate-severe depression and number of condomless sex partners.

## Discussion

We found high prevalence of probable MDD (24%) in this sample of truck drivers. When comparing across studies that also used the PHQ-9 from non-community-based samples, this estimate was higher than a sample of HIV-positive adult men attending clinics in Uganda (12%) (Wagner et al., 2011), but lower than in a study among adult men who have sex with men (MSM) in coastal Kenya enrolled in an HIV research study (42%) (Secor et al., 2015). Nationally representative data on depression in the general population in Kenya are scant. According to the 2003 World Health Survey in Kenya, 5.5% of men reported having been diagnosed with depression in the past 12 months (World Health Survey, 2003). Although we did not evaluate occupational stressors in this study, they may help explain this high prevalence of depression and might have a similar effect on mental health as the stressors reported by other high-risk groups, such as stigma-related abuse reported by MSM (Anderson et al., 2015). Currently, the North Star Alliance does not offer mental health screening, diagnosis, and treatment at their roadside wellness clinics in East Africa; however, the results of this study suggest a need for such services. A next step that should be considered is piloting mental healthcare services integrated with existing HIV prevention and treatment services. Furthermore, it will be important to identify other co-morbidities that can be addressed so as to provide more holistic care to this population.

Truck drivers with moderate-severe depression had roughly 60% more condomless sex partners compared with those with no/minimal depression, after adjusting for demographic variables and number of sex partners. Although we cannot determine whether depression led to an increase in sexual risk behavior because our study was cross-sectional, this finding is consistent with a prospective study among men and women in South Africa, which found that baseline depressive symptomatology was associated with failure to use a condom at last sex during the follow-up interview (Nduna et al., 2010). These findings are supported by Beck’s cognitive theory of depression whereby negative feelings associated with depression can ultimately lead to negative behavioral reactions to situations (Beck, 1995), such as not using a condom when presented with the opportunity for sexual intercourse.

In our mediation analysis, the indirect effect (i.e., the effect of depression on sexual risk behavior that acts through self-efficacy) was statistically significant and the natural direct effect (i.e., the effect of depression on sexual risk behavior unexplained by general self-efficacy) was not statistically significant, thus suggesting self-efficacy as a mediator. In other words, depression might negatively affect an individual’s confidence to cope with stressful situations and perform tasks, and consequently, this lower self-efficacy encourages an individual to engage in higher sexual risk behavior. Self-efficacy is a broad construct, so there may be multiple explanatory pathways between self-efficacy and sexual risk behavior, for example, pathways related to self-esteem, optimism about the future, or belief in the ability to prevent sexually transmitted infections (Kamen et al., 2013). Other studies from sub-Saharan Africa have also found that self-efficacy was a mediator of this association (Wagner et al., 2011; Alvy et al., 2011; Tucker et al., 2013), but have also been cross-sectional. Bandura’s Social Cognitive Theory (Bandura, 1989) might suggest that low self-efficacy could be a cause of depression implicating it as a confounder. A recent study among adolescents examining the bidirectional association found that depression resulted in negative changes in self-efficacy, rather than the other way around (Tak et al., 2017). Thus, further research is needed to understand the role of self-efficacy in this association. If self-efficacy is indeed a mediator, it could also be a target to reduce high sexual risk behavior, especially among individuals with depression, through interventions such as behavioral activation, problem-solving, and exploration of past successes and perceived competencies (Kamen et al., 2013).

There are some limitations to consider when interpreting these data. First, clinic-based sampling alone, as done in our study, may result in higher prevalence estimates for mental health disorders than if non-clinic based sampling was also done (Patten, 2000). However, if the prevalence of depression is higher among those who seek care than those who do not, it would mean that the population with the greatest need for mental healthcare services is perhaps the easiest to reach because they are already in care. Roadside wellness clinics will be an important entry point to initiate the provision of these services to truck drivers. Another limitation related to sampling is that our study participants were recruited from two clinics in one geographical area, so we do not know how similar or different these truck drivers are compared with truck drivers who attend clinics in other areas of Kenya or East Africa. Second, our measure of sexual risk behavior was the number of condomless sex partners, which is not inclusive of all aspects of sexual risk (Aral, 2004). Although condomless sex with only a main partner would be considered lower sexual risk behavior compared with condomless sex with multiple partners, most men in our study had more than one sexual partner, even though most were married, and we adjusted for marital status and number of sex partners in the multivariable model. Third, some variables may have been subject to reporting bias. Because homosexuality is illegal in Kenya, it is understandable why study participants may have been reluctant to discuss same-sex partners. In fact, there was only one participant who reported previously having anal sex with another man. Similarly, participants may have also been reluctant to fully disclose drug and alcohol use because of potential consequences to their livelihood. Third, we did not have data on occupational stressors, which may have acted as confounders or common causes of depression and sexual risk behavior. Finally, as our study was cross-sectional, we do not know the direction of causality for the association between depression and sexual risk behavior. Although the data were interpreted as cross-sectional, measures were taken from two interviews that were 6 months apart, as this was a secondary analysis and the original trial was not specifically designed to answer the research questions in this paper. Because of the limited amount of time truck drivers had for interviews, the PHQ-9 depression scale was administered 6 months later than the questionnaires assessing demographic characteristics, HIV risk behavior, and self-efficacy, so measurement of our exposure (depression) occurred after measurement of our outcome (sexual risk). Although depression status may have changed over the 6 months between the baseline and follow-up interviews, we would not anticipate the association with sexual risk behavior to also change. It is possible that an HIV diagnosis caused depression during follow-up, but only two participants tested positive (Kelvin et al., 2018) and neither of these participants had a PHQ-9 score ≥10. Nevertheless, a longitudinal study is needed to establish the temporal order of events.

Beyond the truck driver population, there are major barriers to accessing mental health services, particularly social stigma and dearth of mental health clinicians (National Academies of Sciences, Engineering, and Medicine, 2016) in Kenya. Although most stigma research in Kenya and sub-Saharan Africa has focused on HIV/AIDS, social stigma regarding mental health disorders appears to be pervasive (Ndetei et al., 2016), but a neglected topic. Individuals might be afraid to seek mental healthcare due to concerns about being labeled and stigmatized by the community, co-workers, or healthcare providers. Because there are a critically low number of mental healthcare providers in Kenya, integrating aspects of mental healthcare in services provided by primary care clinicians and lay health workers must be considered (Jenkins et al., 2010; Marangu et al., 2014). This remains complicated considering the contradictory findings about the impact of providers’ intervention on reducing symptoms of mental health disorders. For example, a trial in Zimbabwe found that lay health workers had a positive impact on reducing symptoms of common mental health disorders through a brief cognitive-behavioral intervention (Chibanda et al., 2016), whereas a trial in Kenya found that a mental health training program for primary care clinicians failed to improve diagnosis of mental health disorders (Jenkins et al., 2013).

## Conclusions

The results of this study suggest an association between depression and sexual risk behavior, and that this association might be mediated by general self-efficacy. Future research is needed to clarify the directionality of these associations. Despite frequent HIV risk-reduction counseling at the roadside clinics included in this study, the prevalence of sexual risk behavior in our participant population was high, emphasizing the importance of bolstering risk-reduction strategies. The prevalence of depression was also high, which emphasizes the importance of developing strategies to improve mental health diagnosis and treatment in this setting. For long-distance truck drivers specifically, future research in sub-Saharan Africa should identify occupational risk factors for depression and other mental health conditions to ensure that evidence-based interventions can be better tailored to this population.

## Supplemental Information

10.7717/peerj.7253/supp-1 Supplemental Information 1 SAS code to recreate analysis.

10.7717/peerj.7253/supp-2 Supplemental Information 2 Study questionnaire.

## Additional Information and Declarations

### Data Availability

The following information was supplied regarding data availability: Data are available at Harvard Dataverse: Kelvin, Elizabeth A; Mantell, Joanne E, 2018, “Increasing HIV testing demand among Kenyan truck drivers and female sex workers”, DOI 10.7910/DVN/8GVXJY, Harvard Dataverse, V2, UNF:6:vT8Ct3ODFigMm1/pWnQzCw== [fileUNF].
