# Supplementary material for: Depression and sexual risk behavior among long-distance truck drivers at roadside wellness clinics in Kenya
Source: PeerJ. 2019 Jul 18;7:e7253. doi: 10.7717/peerj.7253 (PMC6642802; doi:10.7717/peerj.7253)
Supplement: Supplemental Information 2 [file peerj-07-7253-s002.pdf]

# ♦HIV Testing among Truck Drivers♦
# (UpimajiwaVirusivyaUkimwimiongonimwaMaderevaWamalori)
# Baseline Questionnaire
# (MaswaliMsingi)

## A. Administrative Items (MAMBO TEULE

1.DATE (Tarehe): __ __ / __ __ / __ __ __ __ (day, month, year) (siku, mwezi, mwaka)

2.INTERVIEWER NAME (JINA: LA MHOJI):____________________

3. CLINIC (Kliniki): ○ 1=Salgaa○ 2=MaaiMahiu

4. DATA ENTERER'S NAME (JINA LA MWANDIKAJI WA MAMBO YA HAKIKA):_________________

5. STUDY ID # (NAMBARI YA USAJILI YA UTAFITI): _____ _____ _____ _____

6. LANGUAGE OF INTERVIEW (LUGHA YA MAHOJIANO):

○1=English mostly (Kingerezahasa)○2=Kiswahili mostly (Kiswahili hasa)

○3=Bothequally (Yotekwausawa)

NOTE (KUMBUKA): ○=check only one response option (Weka alamakatikajibumojalililochaguliwa);

☐=check all that apply (can check multiple response options) (Weka alamakatikazoteipasavyo

(Unawezakutiaalamamajibumbalimbalizilizochaguliwa)

## B. Demographic Information (Idadi ya Habari)

1.How old were you on your last birthday?(Ulikuwa miaka mingapi wakati uliposheherekea tarehe ya mwisho ya kuzaliwa?)

____ ____ Years(Miaka)*[If participant is unsure, ask for his best guess*(Kama mshiriki hana uhakika, umwulize akujulishe miaka anazotarajia kuwa nazo)*]*

2. What is your religion? (Dini yako ni gani?)

○ 1=Protestant (Kiprotestanti) ○ 2=Catholic (Kikatoliki) ○ 3=Muslim (Kiislamu )

○ 4=Hindu (Kihindu) ○ 5=Traditional African (Jadi wa Kiafrika )

○ 6=No religion (Sina dini)

○ 7=Other, Specify (nyingine, ELEZA) ______________________________

Use the following codes as needed by writing them in the left-hand margin next to the question to which they apply: (Tumia kodi zifuatazo zinavyohitajika kwa kuziandika katika mkono wa kushoto ipasavyo karibu na na kila swali linalohusika nalo )

**SP**=Skipped question due to Skip Pattern (Aliruka kufuatia mfano uliopeanwa)

**RA**=Participant Refused to Answer the question (Mshiriki alikataa kujibu swali)

**DK**=Participant responded that he Does not Know and would not change this response despite probing (Mshiriki alisema kuwa hajui jibu la swali hili na hawezi kubadilisha mtazamo wake hata akifanyiwa uchunguzi Zaidi)

3. What is the highest level of school that you have completed? (Umehitimu kiwango gani cha elimu?)
○1=No education (Sina elimu elimu)
○2=Some primary school (<Grade 8) (Kiwango Fulani cha shule ya msingi ya shule za msingi (<Daraja 8)
○3=Completed primary school (Grade 8) (Nilikamilisha shule ya msingi (darasa la 8)
○4=Some secondary school (>Grade 8, <Grade 12) (Kiwango Fulani cha shule ya sekondari (Zaidi ya darasa la 8, chini ya Daraja 12)
○5=Completed secondary school (Grade 12) (Nilikamilisha elimu ya sekondari (darasa la 12)
○6=Some tertiary education (>Grade 12), specify program (Nina elimu ya juu (> darasa la 12), eleza mpango wa elimu): ______________________
○7=Tertiary school graduate, specify program (Nimehitimu elimu elimu ya juu,nina shahada, eleza mpango wa elimu) : ______________________________

4. What is your marital status? [*if does not answer #2 or 3, skip to question 5]* (Hali yako ya ndoa [Kama Hajejibu swali la # 2 au 3, ruka hadi swali 5])
○1=Single, never married (sijeoa) ○2=Legally married (Nimefunga ndoa kisheria)
○3=Common law marriage or living with partner (Sheria ya kawaida ya ndoa au kuishi na mpenzi)
○4=Divorced/Separated (Tumetengana na mpenzi)
○5=Widowed (Mjane /nimefiwa na mpenzi)
○6=Other status, Specify (ingine, ELEZA) ____________________

4a. If legally married, common law marriage or living with partner (options 2 or 3 in question 5), how many nights a month do you spend in your home with your spouse/partner, on average? (Kama umefunga ndoa kisheria, sheria ya kawaida ya ndoa au unaishi na mpenzi (chaguo 2 au 3 katika swali 5), ni usiku ngapi katika Mwezi unakaa nyumbani na mpenzi wako / mke wako kwa wastani?)

_____ _____ Nights (Usiku ngapi)

5. How long have you worked as a truck driver? (Umefanya kazi kwa muda gani kama dereva wa malori?)

_____ _____ Years (Miaka), _____ _____ Months (Miezi)

Use the following codes as needed by writing them in the left-hand margin next to the question to which they apply: (Tumia kodi zifuatazo zinavyohitajika kwa kuziandika katika mkono wa kushoto ipasavyo karibu na na kila swali linalohusika nalo )
**SP**=Skipped question due to Skip Pattern (Aliruka kufuatia mfano uliopeanwa)
**RA**=Participant Refused to Answer the question (Mshiriki alikataa kujibu swali)
**DK**=Participant responded that he Does not Know and would not change this response despite probing (Mshiriki alisema kuwa hajui jibu la swali hili na hawezi kubadilisha mtazamo wake hata akifanyiwa uchunguzi Zaidi)

6. What county in Kenya do you live in? (Unaishi katika Kaunti gani nchini Kenya?)

○1=Baringo county (Kaunti ya Baringo kata)
○2=Bomet county (Kaunti ya Bomet)
○3=Bungoma county (Kaunti ya Bungoma)
○4=Busia county (Kaunti ya Busia)
○5=Elgeyo-Marakwet county (Kaunti ya Elgeyo-Marakwet)
○6=Embu county (Kaunti ya Embu)
○7=Garissa county (Kaunti ya Garissa)
○8=Homabay county (Kaunti ya Homabay)
○9=Isiolo county (Kaunti ya Isiolo)
○10=Kajiado county (Kaunti ya Kajiado)
○11=Kakamega county (Kaunti ya Kakamega)
○12=Kericho county (Kaunti ya Kericho)
○13=Kiambu county (Kaunti ya Kiambu)
○14=Kilifi county (Kaunti ya Kilifi)
○15=Kirinyaga county (Kaunti ya Kirinyaga)
○16=Kisii county (Kaunti ya Kisii)
○17=kisumu county (Kaunti ya Kisumu)
○18=Kitui county (Kaunti ya Kitui)
○19=Kwale county (Kaunti ya Kwale)
○20=Laikipia county (Kaunti ya Laikipia)
○21=Lamu county (Kaunti ya Lamu)
○22=Machakos county (Kaunti ya Machakos)
○23=Makueni county (Kaunti ya Makueni)
○24=Mandera county (Kaunti ya Mandera)
○25=Marsabit county (Kaunti ya Marsabit)
○26=Meru county (Kaunti ya Meru)
○27=Migori county (Kaunti ya Migori)
○28=Mombasa county (Kaunti ya Mombasa)
○29=Murang'a county (Kaunti ya Murang'a)
○30=Nairobi city county (Kaunti ya mji wa Nairobi)
○31=Nakuru county (Kaunti ya Nakuru)
○32=Nandi county (Kaunti ya Nandi)
○33=Narok county (Kaunti ya Narok)
○34=Nyamira county (Kaunti ya Nyamira)
○35=Nyandarua county (Kaunti ya Nyandarua)
○36=Nyeri county (Kaunti ya Nyeri)
○37=Samburu county (Kaunti ya Samburu)
○38=Siayacounty Siaya (Kaunti ya Siaya)
○39=Taita county (Kaunti ya Taita)
○40=Tana River county (Kaunti ya Tana River)
○41=Tharaka-Nithi county (Kaunti ya Tharaka Nithi)
○42=Trans Nzoia county (Kaunti ya Trans Nzoia)
○43=Turkana county (Kaunti ya Turkana)
○44=Uasin Gishu county (Kaunti ya Uasin Gishu)
○45=Vihiga county (Kaunti ya Vihiga)
○46=Wajir county (Kaunti ya Wajir)
○47=Westpokot county (Kaunti ya Westpokot)
○48=Other, specify (Kaunti nyingine, TAJA) ___________________________

7. Were you born in the same county where you currently live, a different county in Kenya, or a different country? (Ulizaliwa katika kaunti ambayo unaishi au Kaunti nyingine Kenya ama katika nchi ingine?)

○1=Same county where live (skip to question 8) (Kaunti ambapo kuishi (ruka hadi swali la 8)
○2=Different county in Kenya (Kaunti tofauti nchini Kenya)
○3=Different country, specify (nchi tofauti, TAJA): _______________________________

7a. If you were born in a different place from where you currently live, how long have you been living in your current location? (Kama ulizaliwa katika sehemu tofauti na ambapo unaishi, umeishi katika sehemu ambapo unaishi sasa hivi kwa muda gani?)

______ ______ years and (Miaka na) ______ ______ months (miezi)

8. When you drive the truck for work, are you usually alone or does someone else ride with you? (Wakati unapoenda lori kwa ajili ya kazi, huwa unaendesha peke yako ama uwa unasafiri na mtu mwingine?)

○0=Alone (Peke yangu) ○1=Someone rides with me (Tunaenda pamoja na mtu mwingine)

Use the following codes as needed by writing them in the left-hand margin next to the question to which they apply: (Tumia kodi zifuatazo zinavyohitajika kwa kuziandika katika mkono wa kushoto ipasavyo karibu na na kila swali linalohusika nalo )

**SP**=Skipped question due to Skip Pattern (Aliruka kufuatia mfano uliopeanwa)

**RA**=Participant Refused to Answer the question (Mshiriki alikataa kujibu swali)

**DK**=Participant responded that he Does not Know and would not change this response despite probing (Mshiriki alisema kuwa hajui jibu la swali hili na hawezi kubadilisha mtazamo wake hata akifanyiwa uchunguzi Zaidi)

9. If someone else rides with you, who is that person(s)? [*check all mentioned, probe to ask if anyone else usually rides with participant*] (Kama unaenda na mtu mwingine au watu wengine ni nani huyo ama hawa)? [Weka alama katika yote yaliyotajwa, Uliza kama mtu mwingine huwa anaenda na mshiriki]

☐ 1=Assistant paid to help with loading or driving (Msaidizi ambaye ulipwa kwa kusaidia kuendesha gari au kupakua mzigo)
☐ 2=Wife/main partner (Mke / mpenzi kuu)
☐ 3=Son or daughter (Mwana au binti)
☐ 4=Friend or other relative (Rafiki ama mtu mwingine wa jamii)
☐ 5=Non-main partner (casual partner/road wife) (mpenzi asiye mkuu (mpenzi hasiye wa kawaida / mke wa barabara)
☐ 6=Sex worker or person who provides sexual services in exchange for a ride (Kahaba au mtu ambaye hutoa huduma ya ngono badala ya kulipa nauli)
☐ 7=Other person, specify (Mtu mwingine , ELEZA): ______________________________

10. Do you usually drive the route you drove today? [*If not working today, ask about the most recent route driven*] (Je, huwa unaendesha kupitia barabara ambayo umepitia leo? [Kama hayuko kazini, Uliza njia aliyotumia hivi karibuni])

○ 1=Yes [*Skip to question 11*] (Ndiyo [Ruka kwa swali la 11]) ○ 0=No (Hapana)

10a. If not, what is your usual route? [*If does not have a usual route, put SP*] (Kama sivyo, ni barabara gani wewe hupitia kwa kawaida? [Kama hana njia ya kawaida, weka SP])

From (Kutoka): ______________________ To (Hadi):__________________________

11.In the past month (30 days), how many nights were you away from home, traveling for work? (Katika mwezi moja uliopita (siku 30), ni siku ngapi ulikuwa mbali na nyumbani, kusafiri kwa kazi?)

_____ _____ Nights (Usiku)

12. About how much money do you earn in an average month from driving a truck? [*If responds DK or RA, ask question 12a. If gives an average income, skip to question 13*] (Swali hili linahusu kiasi gani cha fedha unachkupata katika mwezi mwezi moja kutokana na kuendesha gari? [Kama atajibu DK au RA, uliza swali 12a. Kama atatoa mapato ya watani, ruka swali 13])

_____ _____, _____ _____ _____ Kenyan Shillings (Shilingi za Kenya)

12a. Could you tell me if your income is {*Read each range and check the one participant confirms as his income range*]… (Unaweza kuniambia kama mapato yako ni {Soma kila kiwango na utie alama katika kiwango ambacho mshiriki anathibitisha kama mapato yake] ...)
○1=Less than 8,000 Kenyan Shillings? (Chini ya shilingi 8,000 za Kenya?)
○2=Between 8,001 and 16,000 Kenyan Shillings? (Kati ya shilingi 8001 na 16,000 za Kenya?)
○3=Between 16,001 and 24,000 Kenyan Shillings? (Kati ya shilingi 16001 na 24,000 za Kenya?)
○4=Between 24,001 and 50,000 Kenyan Shillings? (Kati ya shilingi 24001 na 50,000 za Kenya?)
○5=More than 50,0001 Kenyan Shillings? (Zaidi ya shilingi 50,0001 za Kenya?)

13. How much money does your family earn in an average month from other sources (e.g. money your wife or others in your household earn, income from farming, renting land or house, etc.)? [*If responds DK or RA, ask question 13a. If gives an average family income, skip to section C*] (Kiasi gani cha fedha ambacho familia yako hupata katika mwezi kutoka kufanya kazi zingine (mfano fedha mkeo au wengine katika familia yako hulipwa, mapato kutokana na kilimo, kukodisha ardhi au nyumba, nk)? [Kama anajibu DK au RA, uliza swali 13a. Kama anatoa wastani ya mapato ya familia, ruka hadi kifungu cha C])

_____ _____, _____ _____ _____ Kenyan Shillings (Shilingi za Kenya)

13a. Could you tell me if your family income is {*Read each range and check the one participant confirms as his income range*]… (Unaweza kuniambia kama mapato ya familia yako ni {Soma kila aina na kuangalia mshiriki mmoja unathibitisha kama mapato mbalimbali yake] ...)
○1=Less than 8,000 Kenyan Shillings? (Chini ya shilingi 8,000 za Kenya?)
○2=Between 8,001 and 16,000 Kenyan Shillings? (Kati shilingi 8001 za shilingi 16,000 za Kenya?)
○3=Between 16,001 and 24,000 Kenyan Shillings? (Kati ya Shilingi 16001 na shilingi 24,000 za Kenya?)
○4=Between 24,001 and 50,000 Kenyan Shillings? (Kati ya shilingi 24001 na shilingi 50,000 za Kenya?)
○5=More than 50,0001 Kenyan Shillings? (Zaidi ya shilingi 50,0001 za Kenya?)

**C. Self-Efficacy[1] (Ubinafsi)**
Now I am going to read you a series of statements. Please tell me how true they are for you. (Sasa Mimi naenda kukusomea mfululizo wa taarifa. Tafadhali nieleze ikiwa ni za ukweli kwako.)

1. I can always manage to solve difficult problems if I try hard enough. (Ninaweza kusimamia na kutatua matatizo magumu ikiwa nitajaribu kwa bidii zaidi.)
○1=Not at all true (Sio ukweli kabisa) ○2=Hardly true (Ni nadra kuwa ukweli)
○3=Moderately true (ukweli kiasi) ○4=Exactly true (ukweli kabisa)

2. If someone opposes me, I can find the means and ways to get what I want. (Kama mtu anapingana ,ninaweza kupata njia ya kupata kile ninachotaka.)
○1=Not at all true (Sio ukweli kabisa) ○2=Hardly true (Ni nadra kuwa ukweli)
○3=Moderately true (ukweli kiasi) ○4=Exactly true (ukweli kabisa)

---

Use the following codes as needed by writing them in the left-hand margin next to the question to which they apply: (Tumia kodi zifuatazo zinavyohitajika kwa kuziandika katika mkono wa kushoto ipasavyo karibu na na kila swali linalohusika nalo )
**SP**=Skipped question due to Skip Pattern (Aliruka kufuatia mfano uliopeanwa)
**RA**=Participant Refused to Answer the question (Mshiriki alikataa kujibu swali)
**DK**=Participant responded that he Does not Know and would not change this response despite probing (Mshiriki alisema kuwa hajui jibu la swali hili na hawezi kubadilisha mtazamo wake hata akifanyiwa uchunguzi Zaidi)

3. It is easy for me to stick to my aims and accomplish my goals. (Ni rahisi kwangu kuzingatia malengo yangu ajili ya kutimiza mahitaji.)
○1=Not at all true (Sio ukweli kabisa) ○2=Hardly true (Ni nadra kuwa ukweli)
○3=Moderately true (ukweli kiasi) ○4=Exactly true (ukweli kabisa)

4. I am confident that I could deal efficiently with unexpected events. (Nina hakika kwamba mimi ninaweza kukabiliana na matukio zisizotarajiwa bila shaka.)
○1=Not at all true (Sio ukweli kabisa) ○2=Hardly true (Ni nadra kuwa ukweli)
○3=Moderately true (ukweli kiasi) ○4=Exactly true (ukweli kabisa)

5. Thanks to my resourcefulness, I know how to handle unforeseen situations. (Ninashukuru kwa ubunifu wangu unaonisaidia kushughulikia mambo yasiyotarajiwa.)
○1=Not at all true (Sio ukweli kabisa) ○2=Hardly true (Ni nadra kuwa ukweli)
○3=Moderately true (ukweli kiasi) ○4=Exactly true (ukweli kabisa)

6. I can solve most problems if I invest the necessary effort. (Ninaweza kutatua matatizo zaidi ikiwa nitatia bidii ipasavyo.)
○1=Not at all true (Sio ukweli kabisa) ○2=Hardly true (Ni nadra kuwa ukweli)
○3=Moderately true (ukweli kiasi) ○4=Exactly true (ukweli kabisa)

7. I can remain calm when facing difficulties because I can rely on my coping abilities. (ninaweza kutulia nikipatwa na matatizo kwa sababuninaweza kutegemea uwezo wangu.)
○1=Not at all true (Sio ukweli kabisa) ○2=Hardly true (Ni nadra kuwa ukweli)
○3=Moderately true (ukweli kiasi) ○4=Exactly true (ukweli kabisa)

8. When I am confronted with a problem, I can usually find several solutions. (Wakati ninakabiliwa na matatizo, ninaweza kupata njia za kutatua.)
○1=Not at all true (Sio ukweli kabisa) ○2=Hardly true (Ni nadra kuwa ukweli)
○3=Moderately true (ukweli kiasi) ○4=Exactly true (ukweli kabisa)

9. If I am in trouble, I can usually think of a solution. (Nikipatwa na shida,kwa kawaida ninapata ufumbizi jinsi ya kutatua.)
○1=Not at all true (Sio ukweli kabisa) ○2=Hardly true (Ni nadra kuwa ukweli)
○3=Moderately true (ukweli kiasi) ○4=Exactly true (ukweli kabisa)

10. I can usually handle whatever comes my way. (Ninaweza kukabiliana na kitu chochte kichach.)
○1=Not at all true (Sio ukweli kabisa) ○2=Hardly true (Ni nadra kuwa ukweli)
○3=Moderately true (ukweli kiasi) ○4=Exactly true (ukweli kabisa)

Use the following codes as needed by writing them in the left-hand margin next to the question to which they apply: (Tumia kodi zifuatazo zinavyohitajika kwa kuziandika katika mkono wa kushoto ipasavyo karibu na na kila swali linalohusika nalo )
**SP**=Skipped question due to Skip Pattern (Aliruka kufuatia mfano uliopeanwa)
**RA**=Participant Refused to Answer the question (Mshiriki alikataa kujibu swali)
**DK**=Participant responded that he Does not Know and would not change this response despite probing (Mshiriki alisema kuwa hajui jibu la swali hili na hawezi kubadilisha mtazamo wake hata akifanyiwa uchunguzi Zaidi)

**D. HIV Testing History & Experiencing (Kupima virusi vya ukimwi Historia na Mapito)**
READ: Now I am going to ask you a few questions about HIV testing. People differ in their views and experiences with HIV testing, and there are no right or wrong answers to these questions. We just want to know what your experiences have been. (SOMA: Sasa ninaenda kukuuliza maswali machache kuhusu kupima virusi vya ukimwi. Watu hutofautiana katika maoni yao na uzoefu na kupima virusi vya ukimwi, na hakuna jibu sahihi au makosa katika majibu ya maswali haya. Tunataka kujua vile upimaji umekua.)

1. Have you ever tested for HIV? (Umewahi kupima Virusi vya ukimwi?)
○ 1=Yes (Ndiyo) ○ 0=No [*Skip to question 9*] (Hapana [Ruka kwa hadi swali la 9])

2. If yes, how many times have you been HIV tested in your lifetime? (Kama ndiyo, ni mara ngapi umepimwa virusi vya ukimwi katika maisha yako?)
______ ______ Times

3. How long has it been since you were last tested for HIV? (Imekuwa muda gani tangu upimwe virusi vya ukimwi?)

_____ _____ Years (Miaka) _____ Months (Miezi)

4. In what kinds of places have you been tested for HIV? Have you ever been test in…*[Read each response, check all that participant indicates he has experienced]* (Ni Katika maeneo gani umepimwa virusi vya ukimwi? Je, umewahi kupimwa katika ... [*Soma kila jibu , Tia alama katika yote ambayo mshiriki amewahi kupitia]*)
☐ 1=In a roadside wellness clinic (Katika kliniki ya barabara)
☐ 2=In an HTC clinic (Katika kliniki ya HTC)
☐3=In a doctor's office or other non-HTC clinic (Katika ofisi ya daktari au nyingine zisizo kliniki ya HTC)
☐4=In a hospital (Katika hospitali)
☐5=In a mobile clinic (Katika kliniki za mkono)
☐6=At your workplace (Katika kazini)
☐7=At your home (Katika nyumba yako)
☐8=Some other place, Specify (Baadhi sehemu nyingine, TAJA) _______________

5. When you tested for HIV, who administered the test? Have you ever been tested by a *[Read each response, check all that participant indicates he has experienced]* (Wakati kupima virusi vya ukimwi, ni nani alikupima? Umewahi kupimwa na [Soma kila jibu , tia alama katika kila jibu ambalo mshiriki amepitia])
☐ 1=HTC counsellor (mshauri wa HTC)
☐ 2=Community health worker (mfanyakazi wa afya katika jamii) ☐ 3=Nurse (Muuguzi)
☐ 4=Doctor (Daktari) ☐5=Other, Specify (mwingine, TAJA) _________________

6. Have you ever taken an HIV test with any of your past or current sex partners where you both got the test results together? (Je, umewahi kupimwa virusi vya ukimwi na wapenzi wako wa awali ama mpenzi wako wa sasa ambapo mliona matokeo pamoja?)
○ 1=Yes (Ndiyo) ○ 0=No (Hapana)

7. Have you ever self-tested for HIV? By self-tested I mean you obtained an HIV testing kit and tested yourself by collecting your own blood or oral sample? {*If no, skip to question 8*] (Je, umewahi kupima virusi vya ukimwi wewe mwenyewe? Kwa kupima mwenyewe ninamaanisha wewe ulichukua kifaa cha kupima na kutoa damu ama kupitia usufi wa mdomo {Kama sivyo , ruka hadi swali la 8])

○ 1=Yes (Ndiyo) ○ 0=No [*Skip to question 8*] (Hapana [Ruka kwa swali la 8])

7a. If yes, was this a rapid HIV test from which you received the test results within a few minutes, or did you have to send the kit away to be processed and read by someone else who then gave you the results via phone or mail? (Kama ni hivyo,ulipima haraka na kupata matokeo katika dakika chache au ulituma kifaa ili matokeo yasomwe na mtu mwingime kasha ukatumiwa kupitia barua pepe au simu?)

○1 = Rapid test kit, read results by self (Kupima kwa haraka na kusoma matokeo kwa binafsi)

○2 = Sent test kit away and received results later (Nilituma chombo cha kupima mahali pengine na nikapokea matokeo baadaye)

8. Overall, how would you rate your experience with HIV testing? Would you say very positive, somewhat positive, somewhat negative, very negative, or neutral, which is somewhere in between negative and positive? [*Skip to question 10*] (Kwa ujumla, unaweza kuelezea aje uzoefu wa kupima virusi vya ukimwi? Unaweza kusema ni mazuri sana, ni chanya kiasi fulani, ni hasi Fulani kiasi fulani, hasi sana, au unaegemea upande wowote, ambayo ni mahali chanya au hasi kwa kiwango fulani? [Ruka kwa hadi swali la 10])

○ 1=Very positive (chanya sana) ○ 2=Somewhat positive (chanya kiasi fulani)

○ 3=Neutral (unaegemea upande wowote) ○ 4=Somewhat negative (Hasi kiasi fulani)

○ 5=Very Negative (Hasi sana)

9. Why have you never tested for HIV? (Kwa nini hujewai kupimwa virusi vya ukimwi?)

______________________________________________

10. How often do you think someone like you should be tested for HIV? (Unafikiria ni mara ngapi mtu kama wewe anafaa kupimwa virusi vya ukimwi?)

○ 1=Never, HIV testing is not necessary (Hapana, siyo lazima kupimwa)

○ 2=At least once every 3 months (Angalau mara moja kila baada ya miezi 3)

○ 3=At least once every 6 months (Angalau mara moja kila baada ya miezi 6)

○ 4=At least once every year (Angalau mara moja kila mwaka)

○ 5=At least once every 2 years (Angalau mara moja kila baada ya miaka 2)

○ 6=At least once every 5years (Angalau mara moja kila miaka mitano)

○7=At least once every 10 years (Angalau mara moja kila baada ya miaka 10)

○8=At least once in a lifetime (Angalau mara moja maishani)

11. Was HIV testing the main reason you came to the clinic today? [*if yes, skip to* section E] (Je, ulikuja kliniki leo kupimwa virusi vya ukimwi? [Kama ndiyo, ruka kwa swali la E])

○ 1=Yes (Ndiyo) ○ 0=No (Hapana)

12. If not for HIV testing, was the reason [*Read each response and check all that apply*] (Kama siyo kwa ajili ya kupima virusi vya ukimwi ulikuwa sababu [Soma majibu kila mmoja na kutia alama zote zipasavyo])
☐ 1=For an educational program? (Kwa mpango wa elimu?)
☐ 2=Because of symptoms you need diagnosed and treated? (Kwa sababu kuna dalili ambazo zinahitaji kutambuliwa na kutibiwa?)
☐ 3=For follow-up for a health condition for which you are currently being treated? (Kwa kufuatilia hali ya afya ambayo wewe unatibiwa sasa?)
☐ 4=For routine health check-up or screening? (Kwa kufanya uchunguzi wa afya wa kawaida?)
☐ 5=Some other response, Specify generally (Majibu mengine, TAJA) __________________________?

**E. Anticipated HIV Stigma[2] (Unyanyapaa kutokana na virusi vya ukimwi)**
READ: Do you think any of the following things might happen to you, if you were to test positive for HIV and others found out about your HIV status? Do you think you would… (SOMA: Je, unafikiri mambo yoyote yafuatayo yanaweza kukufanyikia, ungepatikana kuwa na virusi vya ukimwi na wengine wamegundua kuhusu hali yako? Je, unafikiri wewe ungefanyiwa mambo haya ...)

1. Be treated badly by health workers (Kuchukuliwa vibaya na wafanyikazi wa afya)
○1 = Ndiyo ○0 = Hapana

2. Lose your job/livelihood (kupoteza kazi yako)
○1 = Ndiyo ○0 = Hapana

3. Be denied care by family if sick (Familia yako kukataa kukutunza ukiwa mgojwa)
○1 = Ndiyo ○0 = Hapana

4. Be rejected by family (Kukataliwa na familia)
○1 = Ndiyo ○0 = Hapana

5. Be treated badly at work or school (Kuchukuliwavibaya kazini au shuleni)
○1 = Ndiyo ○0 = Hapana

6. Be physically abused by your partner (Kunyanyaswa kimwili na mpenzi wako)
○1 = Ndiyo ○0 = Hapana

7. Experience break-up of your relationship (Kuvunjika kwa uhusiano wa kimapenzi na mpezi wako)
○1 = Ndiyo ○0 = Hapana

8. Become a social outcast (Kukataliwa na jamii)
○1 = Ndiyo ○0 = Hapana

9. Lose your friends (Kupoteza marafiki wako)
○1 = Ndiyo ○0 = Hapana

---

Use the following codes as needed by writing them in the left-hand margin next to the question to which they apply: (Tumia kodi zifuatazo zinavyohitajika kwa kuziandika katika mkono wa kushoto ipasavyo karibu na na kila swali linalohusika nalo )
**SP**=Skipped question due to Skip Pattern (Aliruka kufuatia mfano uliopeanwa)
**RA**=Participant Refused to Answer the question (Mshiriki alikataa kujibu swali)
**DK**=Participant responded that he Does not Know and would not change this response despite probing (Mshiriki alisema kuwa hajui jibu la swali hili na hawezi kubadilisha mtazamo wake hata akifanyiwa uchunguzi Zaidi)

**F. Fatalism**[3]

READ: I am going to read a series of statements and want you to tell me how strongly you agree or disagree with each one. You can say that you strongly agree, somewhat agree, neither agree nor disagree, somewhat disagree or strongly disagree [*Show participant Likert scale card F to help him select a response*]. (SOMA: Ninaenda kusoma baadhi ya vifungu na ningependa uniambie vile unakubaliana nazo ama vile haukubaliani na kila mmoja. Unaweza kusema kwamba kukubaliana kabisa, Unakubaliana kiasi fulani, ama haukubaliani wala unakubaliana, unakubaliana kiasi fulani ama haukubaliani kabisa [Onyesha mshiriki kadi ya Likert wadogo F ili imsaidie kuchagua majibu])

1. If someone is meant to get a serious disease, it doesn't matter what kinds of food they eat, they will get that disease anyway. (Kama mtu atapata ugonjwa mbaya, haijalishi ni aina gani ya vyakula wanavyotumia, yeye atapata tu huo ugojwa.)
○1=Strongly agree (Unakubaliana Kabisa) ○2=Somewhat agree (Unakubaliana kiasi Fulani)
○3=Neither agree nor disagree (Unakubaliana wala haukubaliani)
○4=Somewhat disagree (Haukubaliani kiasi Fulani) ○5=Strongly disagree (Haukubaliani kabisa)

2. If someone is meant to get a serious disease, they will get it no matter what they do. (Kama mtu atapata ugonjwa mbaya, haijalishi ni kitu kipi amekifanya atapata tu huo ugojwa.)
○1=Strongly agree (Unakubaliana Kabisa) ○2=Somewhat agree (Unakubaliana kiasi Fulani)
○3=Neither agree nor disagree (Unakubaliana wala haukubaliani)
○4=Somewhat disagree (Haukubaliani kiasi Fulani) ○5=Strongly disagree (Haukubaliani kabisa)

3. If someone gets a serious disease, that's the way they were meant to die. (Kama mtu atapata ugonjwa mbaya, alikuwa amepangiwa kufa kupitia njia hiyo.)
○1=Strongly agree (Unakubaliana Kabisa) ○2=Somewhat agree (Unakubaliana kiasi Fulani)
○3=Neither agree nor disagree (Unakubaliana wala haukubaliani)
○4=Somewhat disagree (Haukubaliani kiasi Fulani) ○5=Strongly disagree (Haukubaliani kabisa)

4. If someone is meant to have a serious disease, they will get that disease. (Kama mtu amenuiwa kupata ugonjwa mbaya, atapata ugonjwa huo ugonjwa.)
○1=Strongly agree (Unakubaliana Kabisa) ○2=Somewhat agree (Unakubaliana kiasi Fulani)
○3=Neither agree nor disagree (Unakubaliana wala haukubaliani)
○4=Somewhat disagree (Haukubaliani kiasi Fulani) ○5=Strongly disagree (Haukubaliani kabisa)

5. If someone has a serious disease and gets treatment for it, they will probably still die from it. (Kama mtu anaugua ugonjwa mbaya na apate matibabu yake, bado atakufa kutokana na ugojwa huo.)
○1=Strongly agree (Unakubaliana Kabisa) ○2=Somewhat agree (Unakubaliana kiasi Fulani)
○3=Neither agree nor disagree (Unakubaliana wala haukubaliani)
○4=Somewhat disagree (Haukubaliani kiasi Fulani) ○5=Strongly disagree (Haukubaliani kabisa)

6. If someone was meant to have a serious disease, it doesn't matter what doctors and nurses tell them to do, they will get the disease anyway. (Kama mtu alikuwa amenuiwa kupata ugonjwa mbaya, haijalishi nini madaktari na wauguzi kuwaambia afanye bado ataupata ugojwa huo.)
○1=Strongly agree (Unakubaliana Kabisa) ○2=Somewhat agree (Unakubaliana kiasi Fulani)
○3=Neither agree nor disagree (Unakubaliana wala haukubaliani)
○4=Somewhat disagree (Haukubaliani kiasi Fulani) ○5=Strongly disagree (Haukubaliani kabisa)

7. How long I live is already determined. (Muda ambapo nitaishi tayari umedhamiriwa.)
○1=Strongly agree (Unakubaliana Kabisa) ○2=Somewhat agree (Unakubaliana kiasi Fulani)
○3=Neither agree nor disagree (Unakubaliana wala haukubaliani)
○4=Somewhat disagree (Haukubaliani kiasi Fulani) ○5=Strongly disagree (Haukubaliani kabisa)

8. I will die when I am supposed to die. (Nitakufa ukifika wakati ambapo nilikuwa mimepangiwa kufa.)
○1=Strongly agree (Unakubaliana Kabisa) ○2=Somewhat agree (Unakubaliana kiasi Fulani)
○3=Neither agree nor disagree (Unakubaliana wala haukubaliani)
○4=Somewhat disagree (Haukubaliani kiasi Fulani) ○5=Strongly disagree (Haukubaliani kabisa)

9. My health is determined by fate. (Afya yangu imedhamiriwa kwa hatma.)
○1=Strongly agree (Unakubaliana Kabisa) ○2=Somewhat agree (Unakubaliana kiasi Fulani)
○3=Neither agree nor disagree (Unakubaliana wala haukubaliani)
○4=Somewhat disagree (Haukubaliani kiasi Fulani) ○5=Strongly disagree (Haukubaliani kabisa)

10. My health is determined by something greater than myself. (Afya yangu imedhamiriwa kwa mambo makuu nnisiyoyajua mimi mwenyewe.)
○1=Strongly agree (Unakubaliana Kabisa) ○2=Somewhat agree (Unakubaliana kiasi Fulani)
○3=Neither agree nor disagree (Unakubaliana wala haukubaliani)
○4=Somewhat disagree (Haukubaliani kiasi Fulani) ○5=Strongly disagree (Haukubaliani kabisa)

11. I will get diseases if I am unlucky. (Nitapata magonjwa ikiwa mimi sina bahati.)
○1=Strongly agree (Unakubaliana Kabisa) ○2=Somewhat agree (Unakubaliana kiasi Fulani)
○3=Neither agree nor disagree (Unakubaliana wala haukubaliani)
○4=Somewhat disagree (Haukubaliani kiasi Fulani) ○5=Strongly disagree (Haukubaliani kabisa)

12. My health is a matter of luck. (Afya yangu hutokana na kuwa na bahati.)
○1=Strongly agree (Unakubaliana Kabisa) ○2=Somewhat agree (Unakubaliana kiasi Fulani)
○3=Neither agree nor disagree (Unakubaliana wala haukubaliani)
○4=Somewhat disagree (Haukubaliani kiasi Fulani) ○5=Strongly disagree (Haukubaliani kabisa)

13. How long I live is a matter of luck. (Muda nitakayoishi hutatokana na bahati niliyonayo.)
○1=Strongly agree (Unakubaliana Kabisa) ○2=Somewhat agree (Unakubaliana kiasi Fulani)
○3=Neither agree nor disagree (Unakubaliana wala haukubaliani)
○4=Somewhat disagree (Haukubaliani kiasi Fulani) ○5=Strongly disagree (Haukubaliani kabisa)

Use the following codes as needed by writing them in the left-hand margin next to the question to which they apply: (Tumia kodi zifuatazo zinavyohitajika kwa kuziandika katika mkono wa kushoto ipasavyo karibu na na kila swali linalohusika nalo )
**SP**=Skipped question due to Skip Pattern (Aliruka kufuatia mfano uliopeanwa)
**RA**=Participant Refused to Answer the question (Mshiriki alikataa kujibu swali)
**DK**=Participant responded that he Does not Know and would not change this response despite probing (Mshiriki alisema kuwa hajui jibu la swali hili na hawezi kubadilisha mtazamo wake hata akifanyiwa uchunguzi Zaidi)

14. I will stay healthy if I am lucky. (Ni bahati kwangu kuwa na afya nzuri.)
○1=Strongly agree (Unakubaliana Kabisa) ○2=Somewhat agree (Unakubaliana kiasi Fulani)
○3=Neither agree nor disagree (Unakubaliana wala haukubaliani)
○4=Somewhat disagree (Haukubaliani kiasi Fulani) ○5=Strongly disagree (Haukubaliani kabisa)

15. Everything that can go wrong for me does. (Kila kitu ambayo yanaweza kwenda vibaya kwangu huenda hivyo.)
○1=Strongly agree (Unakubaliana Kabisa) ○2=Somewhat agree (Unakubaliana kiasi Fulani)
○3=Neither agree nor disagree (Unakubaliana wala haukubaliani)
○4=Somewhat disagree (Haukubaliani kiasi Fulani) ○5=Strongly disagree (Haukubaliani kabisa)

16. I will have a lot of pain from illness. (Nitakuwa na maumivu mengi kutokana na maradhi.)
○1=Strongly agree (Unakubaliana Kabisa) ○2=Somewhat agree (Unakubaliana kiasi Fulani)
○3=Neither agree nor disagree (Unakubaliana wala haukubaliani)
○4=Somewhat disagree (Haukubaliani kiasi Fulani) ○5=Strongly disagree (Haukubaliani kabisa)

17. I will suffer a lot from bad health. (Nitateseka sana kutokana na afya mbaya.)
○1=Strongly agree (Unakubaliana Kabisa) ○2=Somewhat agree (Unakubaliana kiasi Fulani)
○3=Neither agree nor disagree (Unakubaliana wala haukubaliani)
○4=Somewhat disagree (Haukubaliani kiasi Fulani) ○5=Strongly disagree (Haukubaliani kabisa)

18. I often feel helpless in dealing with the problems of life. (Mara nyingi huwa nahisi mnyonge katika kukabiliana na matatizo ya maisha.)
○1=Strongly agree (Unakubaliana Kabisa) ○2=Somewhat agree (Unakubaliana kiasi Fulani)
○3=Neither agree nor disagree (Unakubaliana wala haukubaliani)
○4=Somewhat disagree (Haukubaliani kiasi Fulani) ○5=Strongly disagree (Haukubaliani kabisa)

19. Sometimes I feel that I'm being pushed around in life. (Wakati mwingine ninahisi kwamba ninasukumwa katika maisha.)
○1=Strongly agree (Unakubaliana Kabisa) ○2=Somewhat agree (Unakubaliana kiasi Fulani)
○3=Neither agree nor disagree (Unakubaliana wala haukubaliani)
○4=Somewhat disagree (Haukubaliani kiasi Fulani) ○5=Strongly disagree (Haukubaliani kabisa)

20. There is really no way I can solve some of the problems I have. (Kwa kweli hakuna njia nitakayotumia kutatua baadhi ya matatizo yanayonikumba maishani.)
○1=Strongly agree (Unakubaliana Kabisa) ○2=Somewhat agree (Unakubaliana kiasi Fulani)
○3=Neither agree nor disagree (Unakubaliana wala haukubaliani)
○4=Somewhat disagree (Haukubaliani kiasi Fulani) ○5=Strongly disagree (Haukubaliani kabisa)

Use the following codes as needed by writing them in the left-hand margin next to the question to which they apply: (Tumia kodi zifuatazo zinavyohitajika kwa kuziandika katika mkono wa kushoto ipasavyo karibu na na kila swali linalohusika nalo )
**SP**=Skipped question due to Skip Pattern (Aliruka kufuatia mfano uliopeanwa)
**RA**=Participant Refused to Answer the question (Mshiriki alikataa kujibu swali)
**DK**=Participant responded that he Does not Know and would not change this response despite probing (Mshiriki alisema kuwa hajui jibu la swali hili na hawezi kubadilisha mtazamo wake hata akifanyiwa uchunguzi Zaidi)

**G. Sexual Behaviour (**Tabia ya Kimapenzi/ngono)

*READ:* People differ in their sexual practices. Some people like to experiment with different types of partners and sexual experiences while other tend to prefer to stick with specific partner types and sexual experiences. I am going to ask you a few questions about your sexual history. I know that sometimes it is embarrassing to talk about sex, but you should know that you can tell me anything and you will not be judged and your answers will be kept completely confidential. (SOMA: Watu hutofautiana katika utendaji wao wa ngono.Watu wengine hupenda kushiriki ngono na wapenzi tofautitofauti wakati mwingine huwa wanapendelea kuwa na mpenzi moja.Nitakuuliza maswali machache kuhusu historia yako ya ngono. Najua kwamba wakati mwingine ni jambo la aibu kujadiliana kuhusu ngono, lakini unapaswa kujua kwamba unaweza kuniambia chochote na huwezi kuhukumiwa na majibu yako yatawekwa kwa siri kabisa.)

1. Have you ever had vaginal sex with a woman? By vaginal intercourse I mean when you put your penis intoa woman's vagina. (Umewahi kufanya ngono ya uke na mwanamke? Ngono ya uke inamaanisha kuweka uume wako kwenye uke wa mwanamke.)

○ 1=Yes (Ndiyo) ○ 0=No (Hapana)

2. Have you ever had anal sex with a man or a woman? By anal sex I mean when you insert your penis into the anus of another man or a woman or another man inserts his penis into your anus. (Umewahi kushiriki ngono kupitia sehemu ya haja kubwa na mwanaume au mwanamke? Ngono ya kupitia sehemu ya haja kubwa inamaanisha kuingiza uume wako katika njia ya haja kubwa ya mtu mwingine au mwanamke au mwanaume mwingine kuingiza uume wake katika njia ya haja kubwa yako.)

○ 1=Yes (Ndiyo) ○ 0=No (Hapana)

2a. If yes, was that with female partners, male partners or both male and female partners? (Ikiwa umefanya hivyo, ulishiriki na wapenzi wa kike au wapenzi wa kiume?ama ulishiriki na wanawake na wanaume wote pamoja?)

○ 1=female partners only (wapenzi kike peke yao) ○ 2=Male partners only (Wapenzi wa kiume)
○ 3=Both male and female partners (Wote wa kiume na wa kike)

3. In your lifetime, how many different women have you had vaginal or anal sex with? [*Ask even if answered no to questions 1 and 2 as a double check*] (Katika maisha yako umewahi kushiriki ngono kupitia njia ya haja kubwa au uke na wanawake wangapi tofauti wanawake kuwa na wewe alikuwa na ngono ya uke au njia ya haja kubwa na? [Uliza hata kama jibu la swali la 1 na2 ni hapana ni wa kutiwa alama mara mbili]

_____ _____ Women [*If zero, ship to question 5*] (Wanawake [Kama hakunai, uliza swali la 5])

4. How old were you the first time you had vaginal or anal sex with a woman? (Ulikuwa wa umri gani mara ya kwanza uliposhiriki ngono ya uke au njia ya haja kubwa na mwanamke?)

(Umri) ______ ______ years old (miaka)

Use the following codes as needed by writing them in the left-hand margin next to the question to which they apply: (Tumia kodi zifuatazo zinavyohitajika kwa kuziandika katika mkono wa kushoto ipasavyo karibu na na kila swali linalohusika nalo )
**SP**=Skipped question due to Skip Pattern (Aliruka kufuatia mfano uliopeanwa)
**RA**=Participant Refused to Answer the question (Mshiriki alikataa kujibu swali)
**DK**=Participant responded that he Does not Know and would not change this response despite probing (Mshiriki alisema kuwa hajui jibu la swali hili na hawezi kubadilisha mtazamo wake hata akifanyiwa uchunguzi Zaidi)

5. In your lifetime, how many different men have you had anal sex with? ? [*Ask even if answered no to questions 1 and 2 as a double check*] (Katika maisha yako, ni wanaume wangapi umewahi kushiriki ngono kupitia njia ya haja kubwa nao? [Uliza hata kama jibu la swali la1 na 2 ni hapana kutia alama mara mbili])

_____ _____ Men [*If answered NO to questions 1 &2 AND answered 0 to questions 3 & 5, skip to question 14]* (Wanaume [Kama alijibu hapana katika maswali 1 na 2 na akajibu 0 maswali katika 3 na 5, ruka hadi swali 14])

6. How old were you the first time you had anal sex with a man? (Ulikuwa wa umri gani mara ya kwanza uliposhiriki ngono kupitia njia ya haja kubwa na mwanamume?)
(Umri) ______ ______ years old (miaka)

7. Have you had vaginal or anal sex with a man or a woman in the past 6 months? [*If no, skip to question 14*] (Je, umeshiriki ngono kupitia uke au kupitia njia ya haja kubwa na mwanamke au mwanamune katika kipindi cha miezi 6 zilizopita? [Akijibu hapana, ruka hadi swali 14])
○ 1=Yes (Ndiyo) ○ 0=No (Hapana)

8. When you had vaginal or anal sex with a man or a woman in the past 6 months, how often did you use a male condom? A male condom is the cover that fits over the man's penis prior to sex. (Wakati uliposhiriki ngono ya uke au kupitia njia ya haja kubwa na mwanamume au mwanamke katika kipindi cha miezi 6, ni mara ngapi ulitumia kondomu ya kiume? kondomu ya kiume ni mpira inayovaliwa katika uume wa mtu kabla ya kushiriki ngono)
○ 1=Always (Kila mara) ○ 2=Sometimes (Wakati mwingine)
○ 3=Never (Sikutumia kamwe)

9. When you had vaginal or anal sex with a man or a woman in the past 6 months, how often did you use a female condom? A female condom is a pouch that is inserted into the vagina or anus prior to sex and the penis goes into the pouch during sex. (Wakati uliposhiriki katika ngono ya uke au kupitia njia ya haja kubwa na mwanamume au mwanamke katika kipindi cha miezi 6, ni mara ngapi ulitumia kondomu za kike? Kondomu ya kike ni mfuko unaoingizwa ndani ya uke au njia ya haja kubwa kabla ya ngono na uume huingizwa katika mfuko huo wakati wa ngono.)

○ 1=Always (Kila mara) ○ 2=Sometimes (Wakati mwingine)
○ 3=Never (Sikutumia kamwe)

10. With how many women have you had vaginal or anal sex with in the past 6 months? (Umeshiriki ngono wa uke au wa kupitia njia ya haja kubwa na wanawake wangapi katika kipindi cha miezi 6?) _______ _____ Women [*If 0, double check and then skip to question 11*] (Wanawake [Kama hakuna, tia alama mara mbili na kisha uruke hadi swali la 11])

11. With how many of those women did you have vaginal or anal sex without a condom (male or female)in the past 6 months, even just one time? (Ni wangapi kati ya wanawake hawa mlishiriki ngono ya uke au kupitia njia ya haja kubwa bila kutumia mpira ya kondomu (mwanamume au mwanamke) katika kipindi cha miezi 6, hata ikiwa ni mara moja pekee) ______ _____ Women (Wanawake)

---

Use the following codes as needed by writing them in the left-hand margin next to the question to which they apply: (Tumia kodi zifuatazo zinavyohitajika kwa kuziandika katika mkono wa kushoto ipasavyo karibu na na kila swali linalohusika nalo )
**SP**=Skipped question due to Skip Pattern (Aliruka kufuatia mfano uliopeanwa)
**RA**=Participant Refused to Answer the question (Mshiriki alikataa kujibu swali)
**DK**=Participant responded that he Does not Know and would not change this response despite probing (Mshiriki alisema kuwa hajui jibu la swali hili na hawezi kubadilisha mtazamo wake hata akifanyiwa uchunguzi Zaidi)

12. How many of these women had sex with you in the past 6 months because you paid them or gave them something like gifts, food or a ride? (Ni wangapi kati ya wanawake hawa mlishiriki kitendo cha ngono katika kipindi cha miezi 6 kwa sababu uliwalipa au kuwapa kitu kama zawadi, chakula au mlisafiri nao?)
_____ _____ Women (Wanawake)

13. With how many men have you had anal sex with in the past 6 months? (Umeshiriki kitendo cha ngono kupitia njia ya haja kubwa katika kipindi cha miezi 6?) _______ _____ Men [*If 0, double check then skip to question 14*] (Wanaume [Ikiwa hakuna tia alama mara mbili kasha uruka hadi swali la 14])

14. With how many of those men did you have anal sex without a condom (male or female) in the past 6 months, even just one time? (Ni wangapi kati ya wanaume hawa mlifanya kitendo cha ngono kupitia njia ya haja kubwa bila kondomu (mwanamume au mwanamke) katika kipindi cha miezi 6, hata ikiwa ni mara mmoja tu?) ______ _____ Men (Wanaume)

15. How many of these men had sex with you in the past 6 months because you paid them or gave them something like gifts, food or a ride? (Ni wanaume wangapi kati yao ambao walishiriki ngono nawe katika kipindi cha miezi 6 kwa sababu uliwalipa au uliwapa kitu kama zawadi, chakula au mlisafiri pamoja?)
_____ _____ Men (Wanaume)

16. Men who travel for work often develop long-term romantic relationships with women or men in the towns through which they travel. By romantic relations I mean romantic attachments that may include vaginal or anal sex or other sexual behaviours such as oral sex or mutual masturbation. Do you have any regular partners, men or women with whom you have romantic relations, other than a wife or main partner at home, who you see on a regular basis, such as when traveling through their town on your regular route? [*If no, double check and then skip to question 18*] (Wanaume wanaosafiri kwa ajili ya kazi mara nyingi huendeleza uhusiano wa kimapenzi kwa muda mrefu na wanawake au wanaume katika miji ambayo wao husafiri. Mahusiano ya kimapenzi kumaanisha kushiriki ngono ya uke au kupitia njia ya haja kubwa au tabia nyingine ya ngono kama vile ngono ya mdomo au punyeto . Je, kuna washirika wowote wa kawaida, wanaume au wanawake ambao una mahusiano ya kimapenzi, isipokuwa mke au mpenzi kuu kule nyumbani, ambao unaweza kuona mara kwa mara, kama vile wakati unaposafiri kupitia mji wao au katika njia yako ya kawaida? [Kama hakuna, tia alama mara mbili kisha ruka hadi swali la 18])
○ 1=Yes (Ndiyo) ○ 0=No (Hapana)

17. If yes, how many regular female partners other than a wife or main partner at home do you have? (Kama kunao, ni wanawake wangapi unaoshiriki nao mara kwa mara ijapokuwa mke wako au mpenzi mkuu kule nyumbani?)
_____ _____ Female partners (Washirika wa kike)

18. If yes, how many regular male partners other than a main partner at home do you have? (Kama kunao, ni wanaume wangapi unaoshiriki mara kwa mara ijapokuwa mpenzi kuu nyumbani?)
_____ _____ Male partners (Washirika Kiume)

---

Use the following codes as needed by writing them in the left-hand margin next to the question to which they apply: (Tumia kodi zifuatazo zinavyohitajika kwa kuziandika katika mkono wa kushoto ipasavyo karibu na na kila swali linalohusika nalo )
**SP**=Skipped question due to Skip Pattern (Aliruka kufuatia mfano uliopeanwa)
**RA**=Participant Refused to Answer the question (Mshiriki alikataa kujibu swali)
**DK**=Participant responded that he Does not Know and would not change this response despite probing (Mshiriki alisema kuwa hajui jibu la swali hili na hawezi kubadilisha mtazamo wake hata akifanyiwa uchunguzi Zaidi)

19. Do you provide financial support to any of these regular partners, such as paying rent for their house, buying them food, or giving them money for household expenses? (Je, unawasaidia kifedha hawa wapenzi wako wa mara kwa mara kama, kama vile kuwalipia kodi ya nyumba kuwanunulia chakula, au kuwapa fedha kwa ajili ya mahitaji mengine?)
○ 1=Yes (Ndiyo) ○ 0=No (Hapana)

20. Thinking about your situation now, what do you think are your chances of getting infected with HIV? Would you say no risk at all, a small risk, a moderate risk, or a great risk? (Katika hali yako sasa, unafikiri unaweza kuambukizwa virusi vya ukimwi? Unaweza kusema hakuna hatari wowote, kuna hatari ndogo, kuna hatari kiasi, au kuna hatari kubwa?)
○ 1=No risk at all (Hakuna hatari wowote) ○2= Small risk (Kuna hatari ndogo)
○ 3=Moderate risk (Kuna hatari kiasi) ○ 0=Great risk (Kunahatari kubwa)

21. Thinking about your situation now, what do you think are your chances of getting infected with a sexually transmitted disease (STI) other than HIV? Would you say no risk at all, a small risk, a moderate risk, or a great risk? (Katika hali yako sasa, unafikiri unaweza kuambukizwa na magonjwa ya zinaa (STI) badala ya virusi vya ukimwi? Unaweza kusema hakuna hatari wowote,kuna hatari ndogo, kuna hatari ya wastani, au kuna hatari kubwa?)
○ 1=No risk at all (Hakuna hatari wowote) ○2= Small risk (Kuna hatari ndogo)
○ 3=Moderate risk (Kuna hatari kiasi) ○0=Great risk (Kunahatari kubwa)

**H. Other Risk Behaviours (Hatazi zingine)**
1. In the past year, how often have you had a drink containing alcohol? (Katika mwaka uliopita, ni mara ngapi umekunywa pombe?)
○1= At least once a day (Angalau mara moja kwa siku)
○2=A few times a week but not every day (mara chache kwa wiki lakini si kila siku)
○3= A few times a month, but not every week (mara chache kwa mwezi, lakini si kila wiki)
○4=a few times a year but not every month (mara chache kwa mwaka lakini si kila mwezi)
○5=Never [*Skip to question 3*] (Sikutumia kamwe [Ruka kwa swali la 3])

2. When you drank alcohol in the past year, how many drinks (cups or glasses_ with alcohol do you usually drink? (Wakati ulipokunywa pombe katika mwaka moja uliopita, ulikunywa kiasi kipi (vikombe au vilasi ngapi kwa kawaida hunywa?)
_____ _____ drinks (Vinywaji)

3. In the past year, how often have you used drugs like marijuana or cocaine? (Katika mwaka uliopita, ni mara ngapi umetumia madawa za kulevya kama bangi au cocaine?)
○1= At least once a day (Angalau mara moja kwa siku)
○2=A few times a week but not every day (mara chache kwa wiki lakini si kila siku)
○3= A few times a month, but not every week (mara chache kwa mwezi, lakini si kila wiki)
○4=a few times a year but not every month (mara chache kwa mwaka lakini si kila mwezi)
○5=Never [*Skip to question 5*] (Sikutumia kamwe [Ruka kwa swali la 5])

4. Have you taken any of these drugs through an injection? (Je, ulipewa dawa hizi kwa kudungwa sindano?)
○ 1=Yes (Ndiyo) ○ 0=No (Hapana)

5. Some men are circumcised, when the skin covering the penis is removed Are you circumcised? (Wanaume wengine wanatahiriwa , wakati ngozi unaofunika uume huondolewa Je,umetahiriwa?)
○ 1=Yes [*Skip to question 7*] (Ndiyo [Ruka hadi swali la 7]) ○ 0=No (Hapana)

6. Are you planning to get circumcised in the future? (Je, una mipango wa kutahiriwa katika siku za baadaye?)
○ 1=Yes (Ndiyo) ○ 0=No [*Skip to section I*] (Hapana [Ruka kwa hadi kifungu cha I])

7. How old were you when you were circumcised? (Ulikuwa na umri gani wakati wewe ulitahiriwa?)
(Umri) _____ _____ years old (miaka)

8. Who circumcised you? (Ni nani alikutahiri?)
○ 1=Traditional healer/circumciser ( mganga wa kale / ngariba )
○ 2= religious leader (e.g. Imam, rabbi) (kiongozi wa dini (kwa mfano Imamu, rabbi))
○ 3=Healthcare provider (nurse, doctor) (Mfanyikazi wa afya (muuguzi, daktari))
○ 4=Other, Specify (mwingine, TAJA): ___________________________

**I. Gender Equitable Men's Scale[4] (Usawa wa jinsia ya wanaume kiwango cha 4)**
READ: Now I am going to read you a series of statements about relationships between men and women. Please tell me if you agree, partially agree, or do not agree with each statement[Show participant Likert scale card I to help him select a response]. (SOMA: Sasa Mimi nitakusomea baadhi ya vifungu kuhusu husiano kati ya wanaume na wanawake. Tafadhali unieleze kama unakubaliana, unakubaliana kidogo, au haukubaliani na kila kauli [Onyesha mshiriki kadi ya Likert ili imsaidie kuchagua majibu].)

1. There are times a woman deserves to be beaten. (Kuna wakati ambapo mwanamke anastahili kupigwa.)
○1=Agree (Ninakubali) ○2=Partially agree (Ninakubaliana kidogo)
○3=Do not agree (Sikubaliani)

2. A woman should tolerate violence in order to keep her family together. (Mwanamke anapaswa kuvumilia vurugu ili kuweka familia yake pamoja.)
○1=Agree (Ninakubali) ○2=Partially agree (Ninakubaliana kidogo)
○3=Do not agree (Sikubaliani)

3. If someone insults a man he should defend his reputation with force if he has to. (Kama mtu ametusi mwanamume anapaswa kutetea heshima yake kwa nguvu.)
○1=Agree (Ninakubali) ○2=Partially agree (Ninakubaliana kidogo)
○3=Do not agree (Sikubaliani)

---

Use the following codes as needed by writing them in the left-hand margin next to the question to which they apply: (Tumia kodi zifuatazo zinavyohitajika kwa kuziandika katika mkono wa kushoto ipasavyo karibu na na kila swali linalohusika nalo )
**SP**=Skipped question due to Skip Pattern (Aliruka kufuatia mfano uliopeanwa)
**RA**=Participant Refused to Answer the question (Mshiriki alikataa kujibu swali)
**DK**=Participant responded that he Does not Know and would not change this response despite probing (Mshiriki alisema kuwa hajui jibu la swali hili na hawezi kubadilisha mtazamo wake hata akifanyiwa uchunguzi Zaidi)

4. It is okay for a man to hit his wife if she won't have sex with him. (Ni sawa kwa mwanamume kumgonga mkewe ikiwa hatashiriki katika kitendo cha ngono naye.)
○1=Agree (Ninakubali) ○2=Partially agree (Ninakubaliana kidogo)
○3=Do not agree (Sikubaliani)

5. A man using violence against his wife is a private matter that shouldn't be discussed outside the couple. (Mtu akivurugana na mkewe ni suala la siri ambalo halipaswi kujadiliwa nje ya wanandoa.)
○1=Agree (Ninakubali) ○2=Partially agree (Ninakubaliana kidogo)
○3=Do not agree (Sikubaliani)

6. It is alright for a man to beat his wife if she is unfaithful. (Ni sawa ikiwa mwanaume atampiga mke wake kwa kutoaminika.)
○1=Agree (Ninakubali) ○2=Partially agree (Ninakubaliana kidogo)
○3=Do not agree (Sikubaliani)

7. It is a woman's responsibility to avoid getting pregnant. (Ni wajibu wa mwanamke kuepuka kupata mimba.)
○1=Agree (Ninakubali) ○2=Partially agree (Ninakubaliana kidogo)
○3=Do not agree (Sikubaliani)

8. A man should be outraged if his wife asks him to use a condom. (Inakubalika kwa mwanaume kukasirika mke wake akimwuliza watumie mpira ya kondomu.)
○1=Agree (Ninakubali) ○2=Partially agree (Ninakubaliana kidogo)
○3=Do not agree (Sikubaliani)

9. Women who carry condoms on them are easy. (Wanawake ambao hubeba kondomu ni rahisi.)
○1=Agree (Ninakubali) ○2=Partially agree (Ninakubaliana kidogo)
○3=Do not agree (Sikubaliani)

10. Only when a woman has a child is she a real woman. (Mwanamke akipata mtoto yeye ni mwanamke halisi.)
○1=Agree (Ninakubali) ○2=Partially agree (Ninakubaliana kidogo)
○3=Do not agree (Sikubaliani)

11. A real man produces a male child. (A mtu halisi inazalisha mtoto wa kiume.)
○1=Agree (Ninakubali) ○2=Partially agree (Ninakubaliana kidogo)
○3=Do not agree (Sikubaliani)

12. It disgusts me when I see a man acting like a woman. (Ninaudhika nikiona mwanamume akionyesha tabia za wanawake.)
○1=Agree (Ninakubali) ○2=Partially agree (Ninakubaliana kidogo)
○3=Do not agree (Sikubaliani)

---

Use the following codes as needed by writing them in the left-hand margin next to the question to which they apply: (Tumia kodi zifuatazo zinavyohitajika kwa kuziandika katika mkono wa kushoto ipasavyo karibu na na kila swali linalohusika nalo )
**SP**=Skipped question due to Skip Pattern (Aliruka kufuatia mfano uliopeanwa)
**RA**=Participant Refused to Answer the question (Mshiriki alikataa kujibu swali)
**DK**=Participant responded that he Does not Know and would not change this response despite probing (Mshiriki alisema kuwa hajui jibu la swali hili na hawezi kubadilisha mtazamo wake hata akifanyiwa uchunguzi Zaidi)

13. A woman should not initiate sex. (Mwanamke hapaswi kuanzisha ngono.)
○1=Agree (Ninakubali) ○2=Partially agree (Ninakubaliana kidogo)
○3=Do not agree (Sikubaliani)

14. You don't talk about sex, you just do it. (Haupaswi kuzungumzia ngono, wewe unafanya tu.)
○1=Agree (Ninakubali) ○2=Partially agree (Ninakubaliana kidogo)
○3=Do not agree (Sikubaliani)

15. A woman who has sex before she marries does not deserve respect. (Mwanamke ambaye ameshiriki mapenzi kabla ya kuolewa hastahili heshima.)
○1=Agree (Ninakubali) ○2=Partially agree (Ninakubaliana kidogo)
○3=Do not agree (Sikubaliani)

16. Men need sex more than women do. (Wanaume wanahitaji kushiriki katika ngono Zaidi yawanawake.)
○1=Agree (Ninakubali) ○2=Partially agree (Ninakubaliana kidogo)
○3=Do not agree (Sikubaliani)

17. Men are always ready to have sex. (Wanaume wako tayari kufanya ngono kila wakati.)
○1=Agree (Ninakubali) ○2=Partially agree (Ninakubaliana kidogo)
○3=Do not agree (Sikubaliani)

18. A man needs other women, even if things with his wife are fine. (Mtu anahitaji wanawake wengine, hata Kama wana uhusiano nzuri na mke wake.)
○1=Agree (Ninakubali) ○2=Partially agree (Ninakubaliana kidogo)
○3=Do not agree (Sikubaliani)

19. It is the man who decides what type of sex to have. (Ni jukumu la mwanamume kuamua aina ya ngono watakaoshiriki.)
○1=Agree (Ninakubali) ○2=Partially agree (Ninakubaliana kidogo)
○3=Do not agree (Sikubaliani)

20. Giving the kids a bath and feeding the kids are the mother's responsibility. (Kuwaosha na kulisha watoto ni jukumu la mama.)
○1=Agree (Ninakubali) ○2=Partially agree (Ninakubaliana kidogo)
○3=Do not agree (Sikubaliani)

21. A woman's most important role is to take care of her home and cook for her family. (Jukumu la muhimu la mwanamke ni kutuza nyumba yake na kupikia familia yake.)
○1=Agree (Ninakubali) ○2=Partially agree (Ninakubaliana kidogo)
○3=Do not agree (Sikubaliani)

22. A man should have the final word on decisions in his home. (Mtu anapaswa kutoa uamuzi katika mambo yote nyumbani kwake.)
○1=Agree (Ninakubali) ○2=Partially agree (Ninakubaliana kidogo)
○3=Do not agree (Sikubaliani)

23. The husband should decide what major household items to buy (Mume anapaswa kuamua vitu vya nyumba vya kununua)
○1=Agree (Ninakubali) ○2=Partially agree (Ninakubaliana kidogo)
○3=Do not agree (Sikubaliani)

24. A woman should obey her husband in all things (Ni lazima mwanamke atii mumewe katika mambo yote)
○1=Agree (Ninakubali) ○2=Partially agree (Ninakubaliana kidogo)
○3=Do not agree (Sikubaliani)

**J. Sensation Seeking[5] (Kuuliza kuhusu Hisia)**
*READ: Now I am going to read a number of statements about attitudes regarding sexual experiences. Let me know how well the statement applies to you. You can say that the statement is not at all like you, that it is not much like you, somewhat like you, or very much like you*. (SOMA: Sasa nitasoma idadi ya kauli kuhusu mitazamo ya kushiriki katika ngono. Ningependa kujua jinsi kauli inatumika yanakuguzia wewe. Unaweza kusema si wakati wote kama mimi, kwamba sio sana kama mimi, kiasi fulani kama mimi, kama mimi sana.)

1. I get bored and restless if I have nothing to do. (Mimi huchoka na kuhangaika ikiwa sina kitu cha kufanya.)
○1 = Not at all like me (Si wakati wote kama mimi)
○2 = Not much like me (Si sana kama mimi)
○3 = Somewhat like me (Kiasi fulani kama mimi)
○4 = Very much like me (kama mimi sana)

2. I sometimes like to do things that are a little frightening. (Wakati mwingine mimi hufanya mambo ya kutisha.)
○1 = Not at all like me (Si wakati wote kama mimi)
○2 = Not much like me (Si sana kama mimi)
○3 = Somewhat like me (Kiasi fulani kama mimi)
○4 = Very much like me (kama mimi sana)

3. I would like to try jumping from high places. (Ningependa kujaribu kuruka kutoka mahali pa juu.)
○1 = Not at all like me (Si wakati wote kama mimi)
○2 = Not much like me (Si sana kama mimi)
○3 = Somewhat like me (Kiasi fulani kama mimi)
○4 = Very much like me (kama mimi sana)

4. I have been known by my friends as a risk taker. (Ninajulikana na marafiki zangu kuwa mimi hushiriki katika mambo yaliyo na hatari.)
○1 = Not at all like me (Si wakati wote kama mimi)
○2 = Not much like me (Si sana kama mimi)
○3 = Somewhat like me (Kiasi fulani kama mimi)
○4 = Very much like me (kama mimi sana)

5. I would enjoy the feeling of jumping off a high cliff into a river below. (Nitafurahia vile nitahisi nikirukia ndani ya mto kutoka nyanda za juu.)
○1 = Not at all like me (Si wakati wote kama mimi)
○2 = Not much like me (Si sana kama mimi)
○3 = Somewhat like me (Kiasi fulani kama mimi)
○4 = Very much like me (kama mimi sana)

Use the following codes as needed by writing them in the left-hand margin next to the question to which they apply: (Tumia kodi zifuatazo zinavyohitajika kwa kuziandika katika mkono wa kushoto ipasavyo karibu na na kila swali linalohusika nalo )
**SP**=Skipped question due to Skip Pattern (Aliruka kufuatia mfano uliopeanwa)
**RA**=Participant Refused to Answer the question (Mshiriki alikataa kujibu swali)
**DK**=Participant responded that he Does not Know and would not change this response despite probing (Mshiriki alisema kuwa hajui jibu la swali hili na hawezi kubadilisha mtazamo wake hata akifanyiwa uchunguzi Zaidi)
